# Perineal squamous cell carcinoma arising from an epidermal cyst: a case report

**DOI:** 10.1186/s12957-018-1442-2

**Published:** 2018-07-28

**Authors:** Byung-Soo Park, Dong Hoon Shin, Soo-Hong Kim, Hyuk Jae Jung, Gyung Mo Son, Hyun Sung Kim

**Affiliations:** 1 Department of Surgery, Pusan National University Yangsan Hospital, 20 Geumo-ro, Mulgeum-eup, Yangsan, Gyungsangnam-do 50612 Republic of Korea; 2 Department of Pathology, Pusan National University Yangsan Hospital, Yangsan, Republic of Korea

**Keywords:** Squamous cell carcinoma, Epidermal cyst, Malignant transformation, Perineum, Excision

## Abstract

**Background:**

Epidermal cysts and squamous cell carcinomas (SCCs) are common skin lesions. However, a malignant change in an epidermal cyst is very rare. The incidence of a malignant change from an epidermal cyst to cutaneous SCC is 0.011–0.045%. In particular, malignant transformation of an epidermal cyst in the perineum is extremely rare. To date, three cases have been reported in the English literature.

**Case presentation:**

We report a case of 51-year-old male with an approximately 15-cm perineal mass. This mass started to grow suddenly 4 months previously and caused great discomfort in the perineum due to the large size. The patient underwent excision of the mass with a negative margin. Histopathological analysis confirmed a microinvasive SCC arising from a proliferating epidermoid cyst.

**Conclusions:**

Even if benign tumors are suspected, a change in size, pain, ulceration, or discharge should indicate the need for surgical resection due to the possibility of a malignant change.

## Background

Epidermal cysts and squamous cell carcinomas (SCCs) are common skin lesions [1]. However, cutaneous SCC arising from an epidermal cyst is quite rare [2]. The most common sites are the head and neck, and it has also been reported in the trunk, limb, and gluteal regions [3]. In particular, SCC arising from an epidermal cyst in the perineum is extremely rare. To the best of our knowledge, three cases have been reported in the English literature until now [1, 4]. Here, we report the case of a perineal cutaneous SCC arising from an epidermal cyst.

## Case presentation

A 51-year-old male was referred to our center due to a large perineal mass. The mass was first discovered 30 years ago as a chestnut-sized small movable cystic nodule. Subsequently, he watched himself for a long time because of no unusual changes. The cyst started to grow suddenly 4 months previous to the visit and caused great discomfort in the perineum due to its large size.

**Fig. 1:**
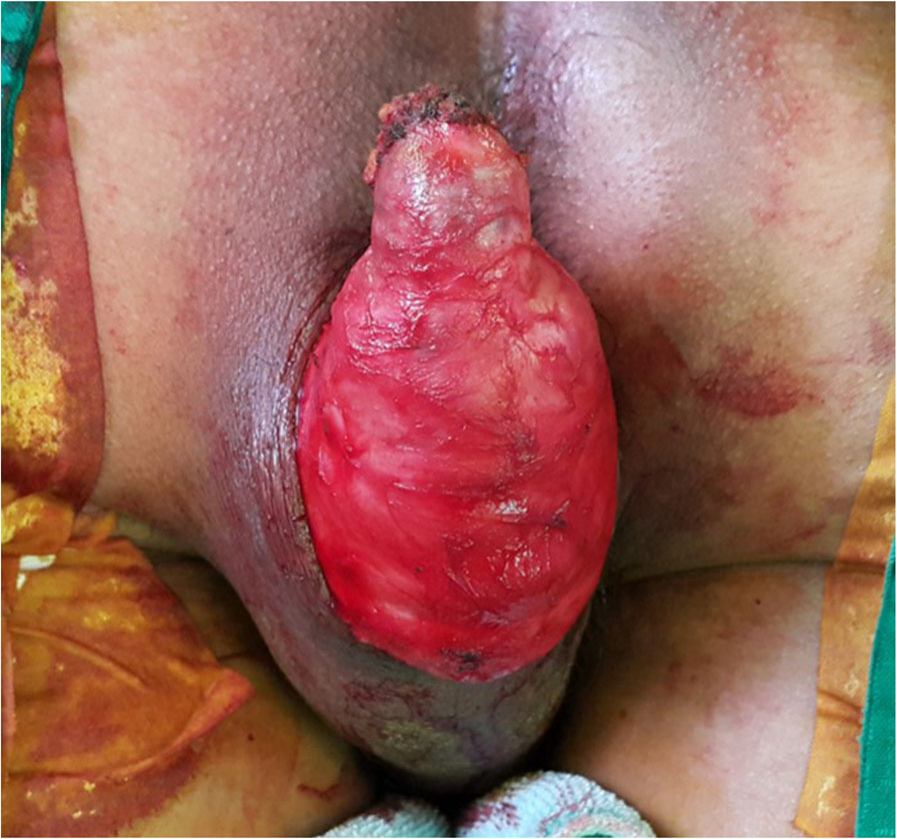
Gross findings in the operative field. An approximately 15-cm cystic mass is in the left perineal area near the anus

On physical examination, an approximately 15-cm cystic mass was observed in the left perineum near the anus. There was no sign of inflammation such as tenderness or redness. Ulceration or discharge was not observed. On a digital rectal examination, there were no specific findings in the anus. A colonoscopy was performed and was unremarkable. Magnetic resonance imaging revealed a 6.7 × 16 cm lobulated mass in the medial aspect of the left perineum with an intermediate signal on T1WI, a high signal on T2WI, and peripheral wall and internal septal enhancement (Fig. 1). There was no significantly enlarged inguinal lymph node. Laboratory values were within normal ranges, except for TPLA (+), FTA-ABS IgG (+), and FTA-ABS IgM (−). Preoperative pathologic tests such as fine needle aspiration and core needle biopsy were not performed because the mass was considered as an epidermal cystic mass.

**Fig. 2:**
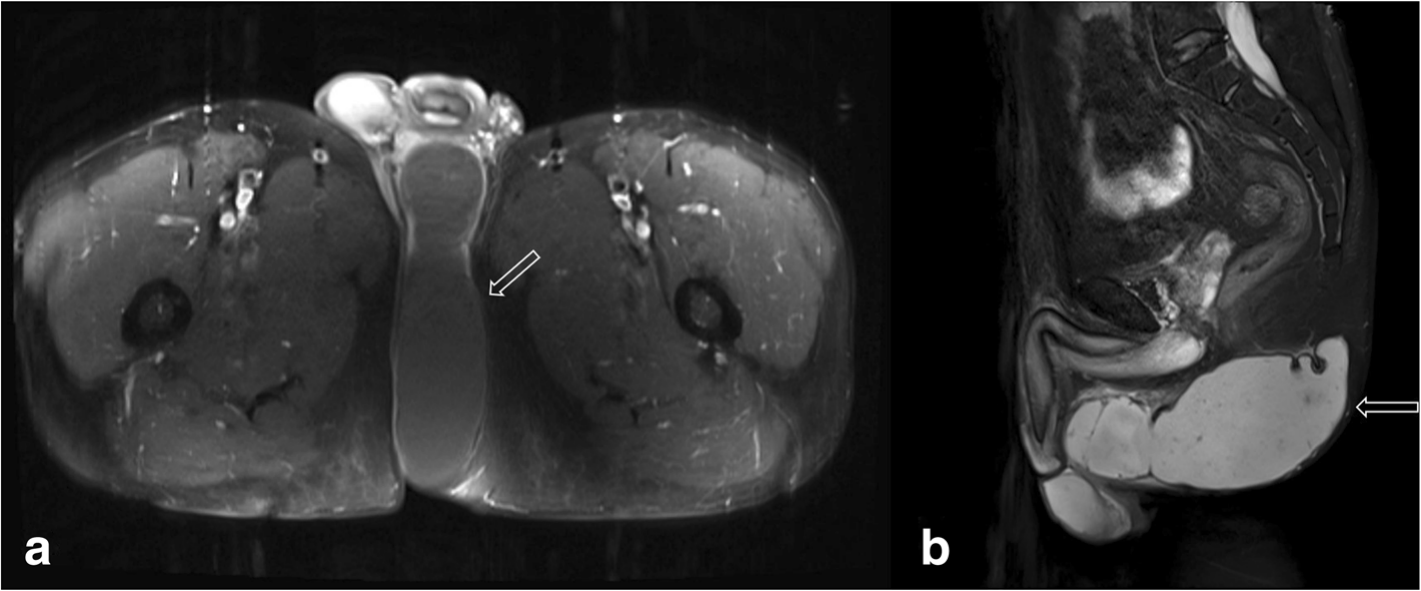
Magnetic resonance imaging findings. The image shows a 6.7 × 16 cm lobulated mass in the medial aspect of the left perineum. **a** Transverse view. **b** Sagittal view

**Fig. 3:**
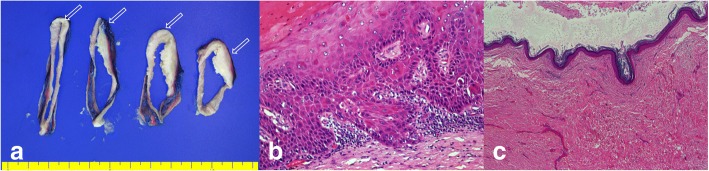
Histopathological findings. **a** Gross findings: microinvasive squamous cell carcinoma (arrow), epidermal cyst wall (thin portion). **b** Microinvasive squamous cell carcinoma (H&E, × 400). **c** Benign cyst (H&E, × 1)

The patient underwent excision of the mass with a negative margin. On exploration, a cystic mass with sebum and keratin was identified in the left perineum (Fig. 2). It had a clear margin without invasion of anal sphincter and urologic tissues. Because the preoperative diagnosis was a cystic mass such as an epidermal inclusion cyst made by the MR pelvis, the surgery was performed with minimal gross margin. The skin was preserved as much as possible and closed easily without any reconstruction. Histopathological analysis showed the cyst had a thin wall composed of benign squamous epithelium. Some sections of the wall were thick, indicating a microinvasive squamous cell carcinoma (pTisN0M0, pStage0) (Fig. 3).

The patient was discharged 2 days after the surgery without a significant postoperative complication. We performed regular follow-up examinations with CT every 6 months, and he showed no evidence of recurrence at 3 years postoperatively.

## Discussion and conclusions

An epidermal cyst is a benign disease caused by invagination of epidermal elements into subcutaneous fat from a hair follicle [5]. Although it is a common skin lesion, malignant transformation of an epidermal cyst is very rare [1, 2]. The incidence of a malignant change from an epidermal cyst to cutaneous SCC is 0.011–0.045% [6]. A recent review of the literature has found 41 well-documented cases of SCC arising from cutaneous epidermal cysts [2]. Most cases have occurred in the head and neck, and cases in trunk, limb, and gluteal tissues have also been reported [2, 3]. In particular, perineal SCC arising from an epidermal cyst is extremely rare, and to date, three cases have been reported in the English literature. In one case each, the lesion was found in the right labia major of a 76-year-old woman [4], in the scrotum of an 86-year-old man in 2013 [7], and in the right vulvar region of a 65-year-old woman in 2016 [1].

The cause of a malignant change in an epidermal cyst is not yet clear. It is known that chronic inflammation or infection can trigger this event, which seems to create dysplasia and/or malignant changes. The most frequent reports are SSCs in the head and neck regions, indicating that the change may be a side effect of ultraviolet radiation. Exposure to ultraviolet radiation and chronic stimuli can be triggered [4, 5]. Although a study was conducted to determine whether human papilloma virus (HPV) can cause malignant transformation of epidermal cyst, the presence of HPV in the lesions was not demonstrated [8]. However, a recent report showed that p16 immunoreactivity was detected in the epidermal cyst wall and accompanying invasive tumors, which is currently used as a surrogate for HPV detection in SCC. Therefore, these authors suggested that SCC arising from an epidermal cyst might be associated with HPV infection [1]. Another possible explanation could be that epidermal cysts contribute to induce local immune destabilization, as observed in other pathological conditions [9]. Further research is still needed on this issue.

Clinically, it is difficult to distinguish between benign cystic lesions and malignant lesions. When an epidermal cyst changes in size or is associated with atypical conditions such as pain, ulceration, or discharge, cancerous transformation should be considered [10]. In the present case, a dramatic change in size was observed. SCC arising from an epidermal cyst should be distinguished from a cystic change of SCC; therefore, for diagnosis, it is essential to show no connection between the tumor and epidermis on a microscopic level [11].

Treatment is not different from that for other skin malignant lesions. Excision with a proper margin is the standard of care. It is recommended that minimal margin of excision be 4 mm, but for high-risk tumors, a 6-mm margin is suggested. Advanced cancer requires extensive surgery with a resection margin of 2 cm or more [12]. The postoperative course is mostly known to have low malignant potential, but there are some reports of an aggressive course, such as metastasis and even mortality [1].

## Conclusion

In conclusion, malignant transformation can occur, even in benign skin lesions. In particular, when there is an atypical situation such as a change in size, pain, ulceration or discharge, surgical resection should be performed due to the possibility of a malignant change. After complete resection, a thorough pathologic examination should be conducted to confirm a malignant transformation.

## Abbreviations

HPV: Human papilloma virus
SCC: Squamous cell carcinoma
